# Senescence promotes in vivo reprogramming through p16^INK^ ^4a^ and IL‐6

**DOI:** 10.1111/acel.12711

**Published:** 2017-12-27

**Authors:** Lluc Mosteiro, Cristina Pantoja, Alba de Martino, Manuel Serrano

**Affiliations:** ^1^ Tumor Suppression Group Spanish National Cancer Research Centre (CNIO) Madrid Spain; ^2^ Institute for Research in Biomedicine (IRB Barcelona) The Barcelona Institute of Science and Technology (BIST) Barcelona Spain; ^3^ Catalan Institution for Research and Advanced Studies (ICREA) Barcelona Spain

**Keywords:** interleukin‐6, p16Ink4a, plasticity, pluripotency, reprogramming, SASP, senescence

## Abstract

Cellular senescence is a damage response aimed to orchestrate tissue repair. We have recently reported that cellular senescence, through the paracrine release of interleukin‐6 (IL6) and other soluble factors, strongly favors cellular reprogramming by Oct4, Sox2, Klf4, and c‐Myc (OSKM) in nonsenescent cells. Indeed, activation of OSKM in mouse tissues triggers senescence in some cells and reprogramming in other cells, both processes occurring concomitantly and in close proximity. In this system, *Ink4a/Arf*‐null tissues cannot undergo senescence, fail to produce IL6, and cannot reprogram efficiently; whereas *p53*‐null tissues undergo extensive damage and senescence, produce high levels of IL6, and reprogram efficiently. Here, we have further explored the genetic determinants of in vivo reprogramming. We report that *Ink4a*, but not *Arf*, is necessary for OSKM‐induced senescence and, thereby, for the paracrine stimulation of reprogramming. However, in the absence of *p53*, IL6 production and reprogramming become independent of *Ink4a*, as revealed by the analysis of *Ink4a/Arf/p53* deficient mice. In the case of the cell cycle inhibitor p21, its protein levels are highly elevated upon OSKM activation in a p53‐independent manner, and we show that *p21*‐null tissues present increased levels of senescence, IL6, and reprogramming. We also report that *Il6*‐mutant tissues are impaired in undergoing reprogramming, thus reinforcing the critical role of IL6 in reprogramming. Finally, young female mice present lower efficiency of in vivo reprogramming compared to male mice, and this gender difference disappears with aging, both observations being consistent with the known anti‐inflammatory effect of estrogens. The current findings regarding the interplay between senescence and reprogramming may conceivably apply to other contexts of tissue damage.

## 1 INTRODUCTION

Senescence is a cellular response to damage characterized by a stable cell cycle arrest and by the secretion of cytokines and other soluble factors with pleiotropic functions, collectively known as senescence‐associated secretory phenotype or SASP (Freund, Orjalo, Desprez & Campisi, 2010; Muñoz‐Espín & Serrano, 2014). The primary role of senescence is thought to be the orchestration of tissue remodeling and repair. This has been demonstrated in a variety of settings, including tissue repair in the skin and liver (Demaria et al., 2014; Krizhanovsky et al., 2008), embryonic development (Davaapil, Brockes & Yun, 2017; Muñoz‐Espín et al., 2013; Storer et al., 2013), placenta formation (Chuprin et al., 2013), as well as cancer regression (Ventura et al., 2007; Xue et al., 2007). In general, senescent cells are efficiently cleared as part of a successful tissue repair process. However, upon severe or chronic damage, senescence‐orchestrated tissue repair may fail and senescent cells may accumulate, contributing to disease and aging (Muñoz‐Espín & Serrano, 2014).

The power of cellular senescence in inducing tissue remodelling has been further extended to processes of cellular reprogramming in vivo. The transgenic expression of the four transcription factors abbreviated as OSKM (Oct4, Sox2, Klf4, and c‐Myc) (Takahashi & Yamanaka, 2006) in adult mice induces dedifferentiation and cellular reprogramming within multiple tissues (Abad et al., 2013; Ohnishi et al., 2014). However, in addition to reprogramming, the activation of OSKM also results in cellular damage and senescence, both in vitro (Banito et al., 2009) and in vivo (Chiche et al., 2017; Mosteiro et al., 2016). Therefore, OSKM induces two opposite cellular fates, namely senescence and reprogramming, that coexist in vivo in separate, but proximal, subsets of cells (Chiche et al., 2017; Mosteiro et al., 2016). Importantly, it has been demonstrated that senescence plays an active role in facilitating in vivo reprogramming through the paracrine action of the SASP, being interleukin‐6 (IL6) a critical mediator (Chiche et al., 2017; Mosteiro et al., 2016). Of note, IL6 plays an important role also during in vitro reprogramming (Brady et al., 2013; Mosteiro et al., 2016). Moreover, the concept that senescence promotes cellular plasticity has been further extended to the activation of somatic stem/progenitor cells. In particular, the SASP can confer somatic stem/progenitor features onto proximal epithelial cells in several tissues (Ritschka et al., 2017).

The tumor suppressor genes *p53*,* p21*,* Ink4a,* and *Arf* act as cell‐autonomous barriers for cellular reprogramming (Banito et al., 2009; Kawamura et al., 2009; Li et al., 2009; Marion et al., 2009; Utikal et al., 2009; Zhao et al., 2008). These barriers are conceivably activated by cellular damages associated to reprogramming, most notably replication stress (Marion et al., 2009), which result in proliferation arrest and, consequently, inhibit reprogramming (Hanna et al., 2009). At the same time, *p53* and the genetic locus *Ink4a/Arf* also affect reprogramming, although in opposite directions, through cell extrinsic mechanisms (Mosteiro et al., 2016). In the absence of *p53*, the induction of OSKM leads to exacerbated damage and senescence in tissues, which results in high levels of IL6 that further enhance reprogramming (Mosteiro et al., 2016). The *Ink4a/Arf* locus plays a complex role in reprogramming: it promotes reprogramming through the paracrine influence of senescence (Mosteiro et al., 2016), and, at the same time, it is a cell‐autonomous barrier for reprogramming (Li et al., 2009). In vivo, the absence of *Ink4a/Arf* severely impairs OSKM‐senescence, IL6 levels are modestly increased, and reprogramming is very inefficient (Mosteiro et al., 2016). Therefore, the positive cell‐autonomous impact of *Ink4a/Arf* deficiency is completely obscured in vivo by the absence of senescence and IL6 secretion (Mosteiro et al., 2016). The emerging picture is that tissue damage and senescence provide a tissue microenvironment that is critical for OSKM reprogramming in vivo.

In this report, we dissect genetically the role of *Ink4a*,* Arf*,* p53, p21,* and *Il6* on in vivo reprogramming.

## 2 RESULTS

### 2.1 *Ink4a* is required for OSKM‐induced senescence and reprogramming

The Ink4a/Arf genetic locus encodes two tumor suppressor genes, namely p16^Ink4a^ and p19^Arf^, acting on separate pathways: p16^Ink4a^ is an inhibitor of the CDK46/cyclin D kinases and, therefore, functions as a positive regulator of the Retinoblastoma family of proteins; p19^Arf^ is an inhibitor of the ubiquitin ligase MDM2, thereby, stabilizing p53 (Gil & Peters, 2006). Previously, we have reported that the activation of an inducible OSKM transgene (abbreviated as *i4F* for inducible four factors) in mice simultaneously lacking *Ink4a* and *Arf* fails to trigger senescence and to produce high levels of IL6, and consequently, reprogramming is severely compromised (Mosteiro et al., 2016). To strengthen the role of IL6 during in vivo reprogramming, we tested whether reprogramming can be rescued in *i4F;Ink4a/Arf*‐null mice by the exogenous administration of recombinant IL6 (rIL6). Of note, we have previously observed that rIL6 administration elevates in vivo reprogramming in wild‐type *i4F* mice (Mosteiro et al., 2016). We focused on the pancreas because it is the tissue that shows the fastest and more widespread senescence, dedifferentiation, and reprogramming (Mosteiro et al., 2016). We observed that *i4F;Ink4a/Arf*‐null mice treated with rIL6 tend to present a higher degree of reprogramming, assessed by the extent of pancreatic dysplasia, upon 7 days of doxycycline treatment (0.2 mg/ml) compared to *i4F; Ink4a/Arf*‐null controls (Figure S1).

To genetically dissect the individual contribution of *Ink4a* and *Arf* during in vivo reprogramming, we generated reprogrammable mouse lines with null alleles for either *Ink4a* (Krimpenfort, Quon, Mooi, Loonstra & Berns, 2001) or *Arf* (Kamijo et al., 1997). After a short treatment with doxycycline, we quantified the following four readouts: the presence of senescent cells (by staining for senescence‐associated β‐galactosidase or SAβG), the mRNA levels of several factors characteristically expressed by senescent cells (collectively known as the SASP), the extent of dysplasia (which we use as a surrogate for cell dedifferentiation and partial reprogramming), and the occurrence of advanced or full reprogramming (by staining for the pluripotency marker NANOG).

**Figure 1:**
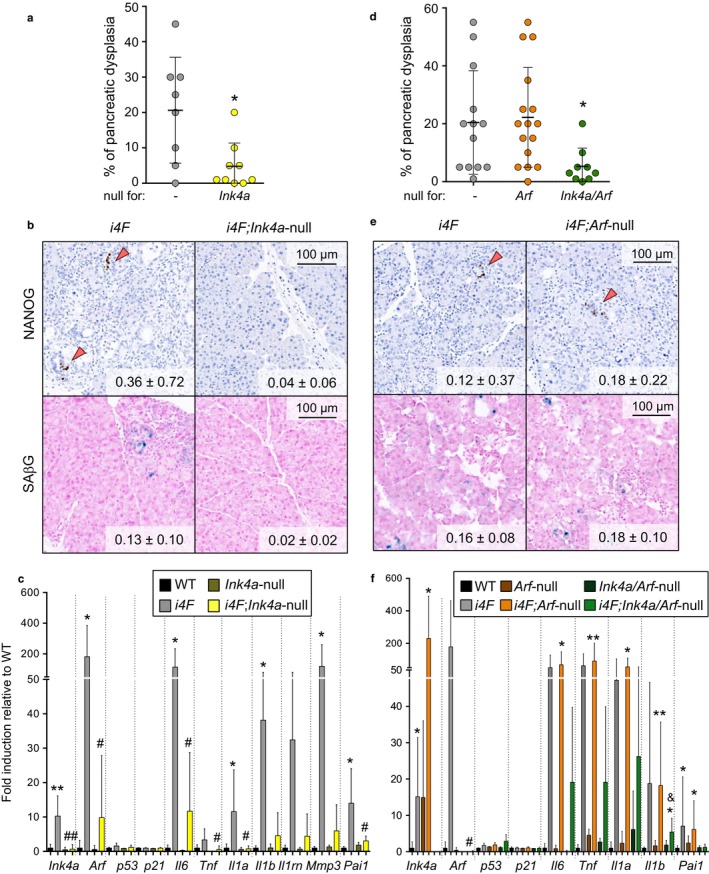
*Ink4a* is required, and *Arf* is dispensable for in vivo OSKM‐induced senescence and reprogramming. a. Percentage of dysplasia in the pancreas of *i4F* and *i4F;Ink4a*‐null mice treated with 0.2 mg/ml doxycycline for 10 days and analyzed at the end of the treatment. Values correspond to the percentage (%) of pancreatic area affected (evaluated blindly). b. NANOG immunohistochemistry and SAβG staining in the pancreas of the same mice as in panel a. Images are representative for at least six mice (*n* ≥ 6) in NANOG and three mice (*n* ≥ 3) in SAβG. Values correspond to ‰ of NANOG+ cells and % of SAβG area quantified with an automated software. c. mRNA levels of the indicated genes in the pancreas of the same mice as in panel a. The number of mice tested is: *n* = 5 in WT and *Ink4a*‐null; *n* = 8 in *i4F*;* n* = 10 in *i4F;Ink4a*‐null. d. Percentage of dysplasia in the pancreas of *i4F*,* i4F;Arf*‐null, *and i4F;Ink4a/Arf*‐null mice treated with 0.2 mg/ml doxycycline for 7 days and analyzed at the end of the treatment. Values correspond to the percentage (%) of pancreatic area affected (evaluated blindly). e. NANOG immunohistochemistry and SAβG staining in the pancreas of the same mice as in panel d. Images are representative for at least 13 mice (*n* ≥ 13) in NANOG and three mice (*n* ≥ 3) in SAβG. Values correspond to ‰ of NANOG+ cells and % of SAβG area quantified with an automated software. f. mRNA levels of the indicated genes in the pancreas of the same mice as in panel d. The number of mice tested is: *n* = 3 in WT,* Arf*‐null and *Ink4a*/*Arf*‐null; *n* = 10 in *i4F*;* n* = 12 in *i4F;Arf*‐null and *n* = 6 in *i4F;Ink4a/Arf*‐null. All the mice tested were males of 8–10 weeks of age. All values are expressed as average ± *SD* Statistical significance compared to WT controls was assessed using the unpaired two‐tailed Student's *t* test with Welch's correction: *p *<* *.05, *; *p *<* *.01, **. Comparisons of each genotype to *i4F* controls are indicated in the same manner but using the symbol “#.” Comparisons between *i4F;Arf*‐null and *i4F;Ink4a/Arf*‐null are indicated in the same manner but using the symbol “&”

We compared *i4F;Ink4a*‐null and *i4F* mice of mixed genetic background, but derived from the same parents and therefore with the same mixed genetic background. After 10 days of doxycycline treatment (0.2 mg/ml), we observed that the degree of pancreatic dysplasia (Figure 1a) and reprogramming (Figure 1b) was strongly decreased in *i4F;Ink4a*‐null mice compared to their *i4F* counterparts. In parallel to this, *i4F;Ink4a*‐null pancreas presented limited senescence after OSKM activation (Figure 1b), and little expression of senescence‐associated genes like *Arf, p53,* and *p21* as well as *Il6* and other factors involved in the SASP, such as *Tnf*,* Il1a*,* Il1b, Il1rn, Mmp3,* and *Pai1* (Figure 1c). Of note, *p21* mRNA was not induced in *i4F* mice (Figure 1c), nor in any of the genetic combinations studied in this work (see later). In relation to this, we have previously reported that upon in vivo OSKM activation, the protein levels of p21 are highly induced in a p53‐independent manner (Mosteiro et al., 2016). These observations suggest that during in vivo reprogramming p21 protein levels are regulated post‐transcriptionally, possibly through one or some of the multiple post‐transcriptional mechanisms that are known to regulate p21 (Scoumanne, Cho, Zhang & Chen, 2011; Warfel & El‐Deiry, 2013; Zhang & Chen, 2008).

Next, we evaluated the extent of *in situ* reprogramming in the pancreas of *i4F;Arf*‐null mice. We compared in parallel the behavior of *i4F*,* i4F;Arf*‐null, and *i4F;Ink4a/Arf*‐null mice, all in a pure C57BL6 genetic background. Interestingly, *i4F;Arf*‐null mice presented similar levels of pancreatic dysplasia compared to *i4F* mice after 7 days of doxycycline treatment (0.2 mg/ml), while *i4F;Ink4a/Arf*‐null pancreatic tissue was almost unaffected (Figure 1d). We also observed that the amounts of NANOG+ cells as well as senescent cells in the pancreas of *i4F;Arf*‐null and *i4F* mice were comparable (Figure 1e). Finally, we confirmed that the expression of SASP factors in *i4F;Arf*‐null pancreas was increased to similar levels as those observed in *i4F* pancreas upon OSKM activation (Figure 1f). All together, these results demonstrate that *Ink4a* is required for OSKM‐induced senescence and secretion of IL6, while *Arf* plays a minimal role in these processes. This is in agreement with our previous data showing that *Il6* and *Tnf* are induced in mouse embryonic fibroblasts (MEFs) upon radiation‐induced senescence and this is abolished when either *Ink4a* or *Ink4a/Arf* are knocked‐down, but not when *Arf* is knocked‐down (Mosteiro et al., 2016).

### 2.2 Absence of *p21* increases OSKM‐induced senescence and reprogramming

**Figure 2:**
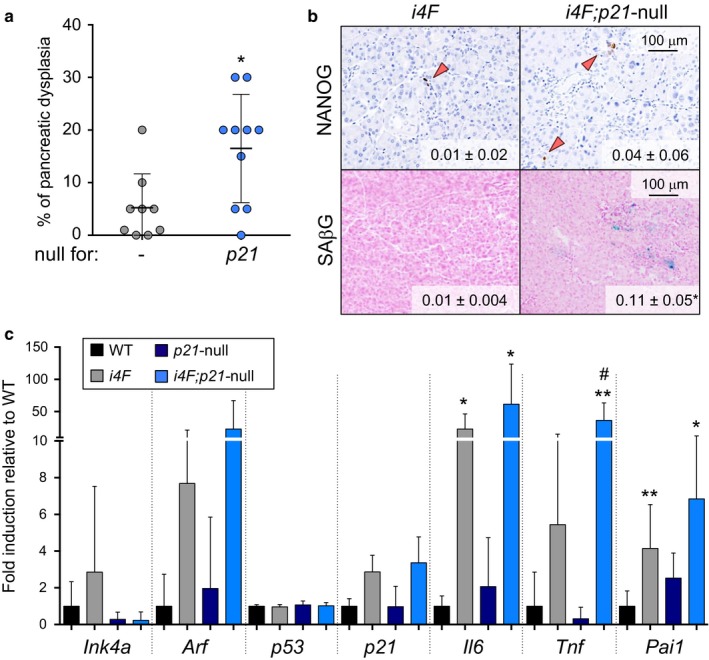
*p21* limits in vivo OSKM‐induced senescence and reprogramming. a. Percentage of dysplasia in the pancreas of *i4F* and *i4F; p21*‐null mice treated with 0.2 mg/ml doxycycline for 7 days and analyzed at the end of the treatment. Values correspond to the percentage (%) of pancreatic area affected (evaluated blindly). b. NANOG immunohistochemistry and SAβG staining in the pancreas of the same mice as in panel a. Images are representative for at least 9 mice (*n* ≥ 9) in NANOG and 3 mice (*n* ≥ 3) in SAβG. Values correspond to ‰ of NANOG+ cells and % of SAβG area quantified with an automated software. c. mRNA levels of the indicated genes in the pancreas of the same mice as in panel a. The number of mice tested is: *n* = 6 in WT;* n* = 8 in *p21*‐null; *n* = 9 in *i4F*; and *n* = 10 in *i4F;p21*‐null. All the mice tested were females of 12–16 weeks of age. All values are expressed as average ± *SD* Statistical significance compared to WT controls was assessed using the unpaired two‐tailed Student's *t* test with Welch's correction: *p *<* *.05, *; *p *<* *.01, **. Comparisons between *i4F* and *i4F;p21*‐null are indicated in the same manner but using the symbol “#”

The cell cycle inhibitor p21^CIP1^ participates in senescence (Muñoz‐Espín & Serrano, 2014; Sharpless & Sherr, 2015), and it is a cell‐autonomous barrier for in vitro reprogramming (Banito et al., 2009; Kawamura et al., 2009). The protein levels of p21 are elevated in tissues expressing OSKM and this occurs independently of p53 (Mosteiro et al., 2016) and, possibly, through post‐transcriptional mechanisms (see above Figure 1c). To investigate the role of p21 during in vivo reprogramming, we generated reprogrammable mice deficient for *p21* (Brugarolas et al., 1995). We first evaluated the reprogramming efficiency in the pancreas of *i4F;p21*‐null mice compared to *i4F* controls, all of pure C57BL6 genetic background, after 7 days of doxycycline treatment (0.2 mg/ml). Interestingly, in the case of *i4F;p21*‐null pancreas, we observed that the degree of pancreatic dysplasia was significantly higher than in *i4F* pancreas (Figure 2a). We also observed an increased accumulation of reprogrammed (NANOG+) and senescent (SAβG+) cells in the pancreas of *i4F;p21*‐null mice compared to *i4F* controls (Figure 2b). In agreement, the levels of SASP factors, such as *Il6*,* Tnf,* and *Pai1*, presented the same tendency, that is, *i4F*<*i4F;p21*‐null (Figure 2c). Therefore, p21 is a negative regulator of OSKM‐induced senescence and reprogramming in vivo.

### 2.3 Absence of *p53* renders IL6 production and reprogramming independent of *Ink4a*

**Figure 3:**
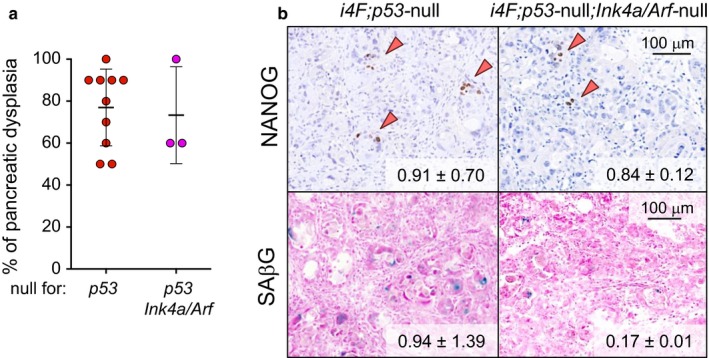
Deletion of *p53* is dominant over the deletion of *Ink4a/Arf* during in vivo OSKM‐induced senescence and reprogramming. a. Percentage of dysplasia in the pancreas of *i4F;p53*‐null and *i4F;p53*‐null;*Ink4a/Arf*‐null mice treated with 0.2 mg/ml doxycycline for 7 days and analyzed at the end of the treatment. Values correspond to the percentage (%) of pancreatic area affected (evaluated blindly). b. NANOG immunohistochemistry and SAβG staining in the pancreas of the same mice as in panel a. Images are representative for at least four mice (*n* ≥ 4) except in *i4F; p53*‐null; *Ink4a/Arf*‐null (*n* = 2). Values correspond to ‰ of NANOG+ cells and % of SAβG area quantified with an automated software. All the mice tested are males of 10–14 weeks of age

We have previously reported that *p53*‐null mice present massive levels of senescence and reprogramming upon OSKM activation, and both processes are partially inhibited by in vivo treatment with anti‐IL6 antibodies (Mosteiro et al., 2016). Given the essential role of *Ink4a* in senescence and IL6 secretion, we investigated its importance in a *p53*‐deficient context and, for this, we generated *p53/Ink4a/Arf* mice. Interestingly, we observed that, after 7 days of doxycycline treatment (0.2 mg/ml), *i4F;p53*‐null;*Ink4a/Arf*‐null mice had a similar degree of dysplasia as *i4F;p53*‐null mice, all being in a pure C57BL6 genetic background (Figure 3a). Similarly, the amount of reprogramming assessed by the presence of NANOG+ cells in the pancreas was comparable between *i4F;p53*‐null and *i4F;p53*‐null;*Ink4a/Arf*‐null mice (Figure 3b). We next evaluated the accumulation of senescent cells and observed that it was decreased *i4F;p53*‐null;*Ink4a/Arf‐*null pancreas compared to *i4F;p53*‐null pancreas (Figure 3b), reinforcing the importance of *Ink4a* in the induction of OSKM‐driven senescence (Mosteiro et al., 2016). However, no significant differences were detected regarding the upregulation of the SASP between *i4F;p53*‐null and *i4F;p53*‐null;*Ink4a/Arf*‐null mice (Figure S2). This indicates that in a *p53*‐null context, the SASP becomes independent of *Ink4a/Arf*. In this regard, it is known that p53 is a negative regulator of the SASP and its deficiency results in a massive upregulation of the SASP (Coppé et al., 2008). Our current observations with *i4F;p53*‐null;*Ink4a/Arf*‐null mice also suggest that the SASP, rather than the extent of senescence, is the key factor responsible for a high reprogramming efficiency. All together, we conclude that the absence of *p53* has a dominant effect over the absence of *Ink4a* with regard to cytokine secretion and this is key in determining the efficiency of reprogramming.

### 2.4 *Il6* mutation impairs in vivo reprogramming

**Figure 4:**
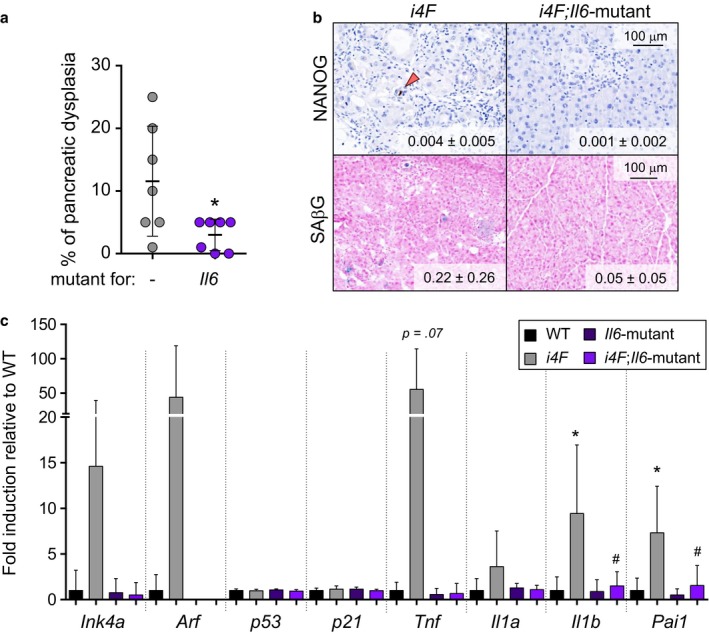
Deletion of *Il6* impairs in vivo OSKM‐induced senescence and reprogramming. a. Percentage of dysplasia in the pancreas of *i4F* and *i4F;Il6‐*mutant mice treated with 0.2 mg/ml doxycycline for 7 days and analyzed at the end of the treatment. Values correspond to the percentage (%) of pancreatic area affected (evaluated blindly). b. NANOG immunohistochemistry and SAβG staining in the pancreas of the same mice as in panel a. Images are representative for at least four mice (*n* ≥ 4) in NANOG and at least three mice (*n* ≥ 3) in SAβG. Values correspond to ‰ of NANOG+ cells and % of SAβG area quantified with an automated software. c. mRNA levels of the indicated genes in the pancreas of the same mice as in panel a. The number of mice tested is *n* = 5 in WT and *Il6‐*mutant and *n* = 7 in *i4F* and *i4F;Il6‐*null. All the mice tested were males of 9–18 weeks of age. All values are expressed as average ± *SD* Statistical significance compared to WT controls was assessed using the unpaired two‐tailed Student's *t* test with Welch's correction: *p *<* *.05, *. Comparisons of each genotype to *i4F* controls are indicated in the same manner but using the symbol “#”

Damaged and senescent cells promote the reprogramming of their neighboring cells through secreted factors, being IL6 one of the key mediators of this cross talk (Mosteiro et al., 2016). Moreover, IL6 plays an important role during in vitro reprogramming (Brady et al., 2013) and treatment with anti‐IL6 antibodies decreases reprogramming both in vitro (Mosteiro et al., 2016) and in vivo (Chiche et al., 2017; Mosteiro et al., 2016), while exogenous administration of rIL6 enhances in vivo reprogramming (Mosteiro et al., 2016) (see also Figure S1). To further strengthen the role of IL6 during in vivo reprogramming, we generated reprogrammable mouse lines (*i4F*) combined with mutant alleles of *Il6* (Kopf et al., 1994). These groups of mice were of mixed genetic background but were derived from the same parents, thereby sharing a comparable genetic background. We observed that after 7 days of doxycycline treatment (0.2 mg/ml), the pancreatic dysplasia in *i4F;Il6*‐mutant mice was much reduced compared to their *i4F* counterparts (Figure 4a). In this genetic background and at the time point analyzed, the number of NANOG+ cells was too low to assess differences between genotypes (Figure 4b), but we observed a suggestive reduction in the levels of senescent cells in the pancreas (Figure 4b) together with a residual induction of *Ink4a*,* Arf*, and several SASP factors, such as *Tnf*,* Il1a*,* Il1b*, and *Pai1* (Figure 4c). This is in agreement with the previously reported role of IL6 in the induction of paracrine senescence (Kuilman et al., 2008). All together, these results reinforce the critical role of IL6 for in vivo reprogramming.

### 2.5 In vivo reprogramming is decreased in female mice

**Figure 5:**
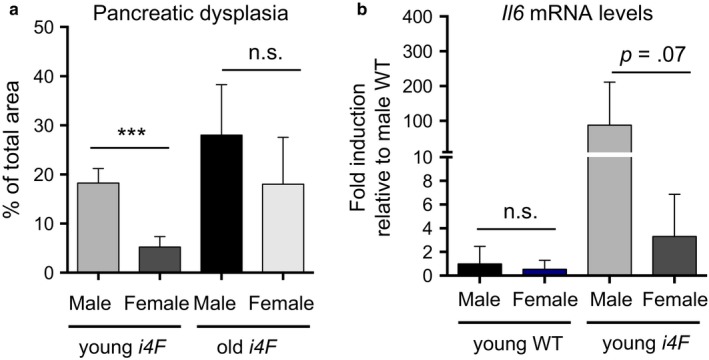
Female mice present reduced in vivo reprogramming. a. Percentage of dysplasia in the pancreas of *i4F* mice from young male (*n* = 28, from Figures 1a,d, and 4a), young female (*n* = 9, from Figure 2a), old male (*n* = 7) and old female (*n* = 5), treated with 0.2 mg/ml doxycycline for 7 days, and analyzed at the end of the treatment. Young male and female mice were 8–12 weeks of age; old male and female mice were ≥54 weeks of age. Values correspond to the percentage (%) of pancreatic area affected (evaluated blindly). b. mRNA levels of the indicated genes in the pancreas of the indicated mice, treated with 0.2 mg/ml doxycycline for 7 days, and analyzed at the end of the treatment. The number of mice tested is *n* = 8 in male WT;* n* = 9 in female WT;* n* = 8 in male *i4F* and *n* = 9 in female *i4F*. All values are expressed as average ± *SD* Statistical significance was assessed using the unpaired two‐tailed Student's *t* test with Welch's correction: *p *<* *.001, ***. Some nonsignificant (n.s.) comparisons are also indicated

All the above analyses were performed using groups of young mice of the same sex, usually males, with the only exception of the experiment shown in Figure 2 that was performed with females. We noted that, upon OSKM induction, control female *i4F* mice presented reduced levels of senescence, dysplasia and reprogramming compared to control *i4F* males. To substantiate this quantitatively, we compiled our current data in young (8–12 weeks of age) *i4F* males from experiments in Figures 1a,d, and 4a and compared them with *i4F* females of the same age from the experiment in Figure 2a. Interestingly, this comparison revealed that young female mice are indeed less prone to undergo reprogramming compared to young male mice (Figure 5a). It is well established that the hormone‐bound form of the estrogen receptor inhibits NκB (Kalaitzidis & Gilmore, 2017; Naugler et al., 2007). NFκB is a major inducer of the SASP and IL6 and, indeed, pharmacological inhibition of NF‐κB reduces OSKM‐induced senescence and reprogramming in vivo (Mosteiro et al., 2016). Therefore, the observed reduced reprogramming of female mice could reflect an attenuated production of IL6 in response to OSKM. To support this, we evaluated the levels of *Il6* mRNA in the pancreas of male and female mice. Interestingly, compared to males, females presented a much lower upregulation of *Il6* upon OSKM activation (Figure 5b).

During aging, there is a general increase in systemic inflammation and IL6 levels, both in males and females (Cevenini, Monti & Franceschi, 2013; Maggio, Guralnik, Longo & Ferrucci, 2006) and, accordingly, we observed that aged mice undergo in vivo reprogramming more efficiently than young mice (Mosteiro et al., 2016). Aging also results in lower levels of estrogens in female mice (Nelson et al., 1981). Based on this, we hypothesized that aged female mice should have an in vivo reprogramming efficiency similar to the one in aged male mice. To address this, we compared the degree of pancreatic dysplasia in a group of old females and males (≥54 weeks of age). Interestingly, the gender differences observed in young mice disappeared in old mice (Figure 5a).

In summary, the gender‐specific difference observed in reprogramming and its attenuation with aging is consistent with the concept that the levels of IL6 strongly influence in vivo reprogramming and suggest that estrogens negatively regulate in vivo reprogramming.

## 3 DISCUSSION

**Figure 6:**
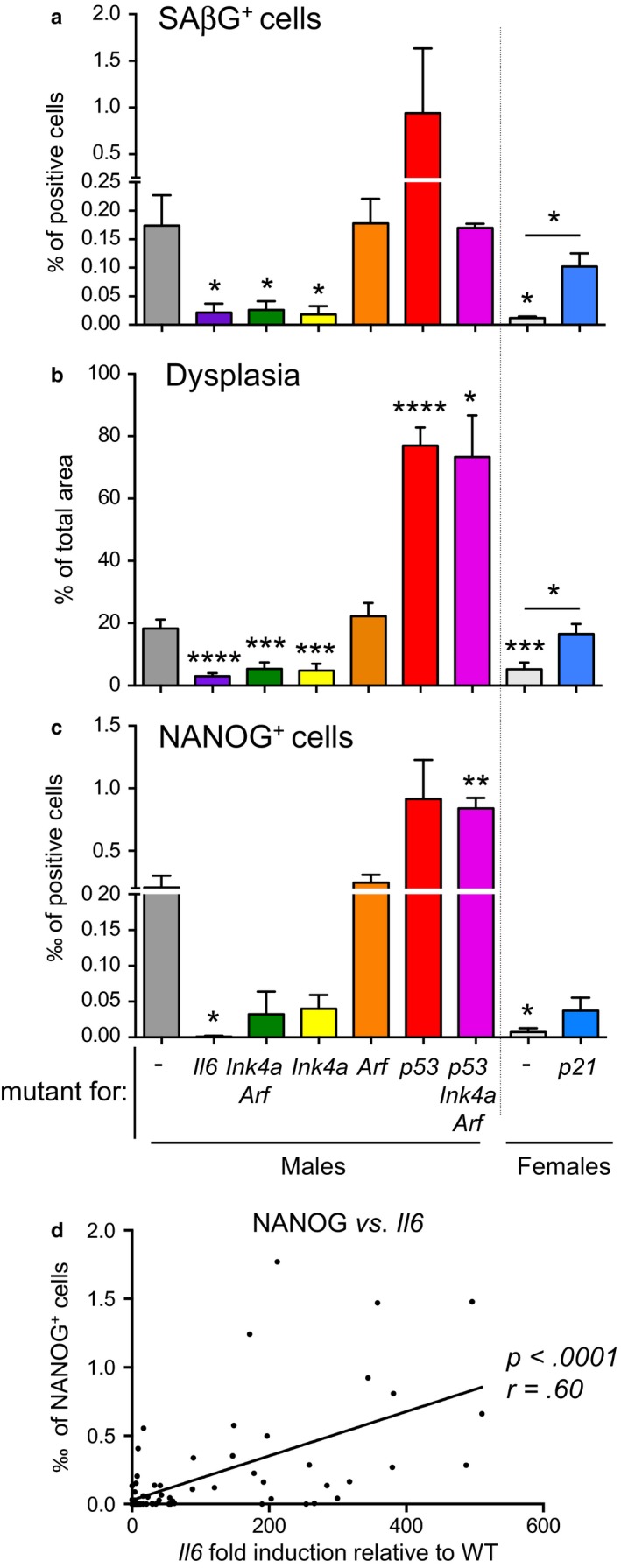
Genetic dissection of OSKM‐induced reprogramming and senescence. a. Compilation of senescence induction (SAβG+ cells) by OSKM in the pancreas of the indicated mice from Figures 1, 2, 3, 4. *N* values are: *i4F*,* n* = 10; *i4F*;*Il6*‐mutant, *n* = 3; *i4F*;*Ink4a/Arf*‐null, *n* = 3; *i4F;Ink4a*‐null, *n* = 3; *i4F;Arf*‐null, *n* = 5; *i4F;p53*‐null, *n* = 4; *i4F;p53*‐null;*Ink4a/Arf*‐null, *n* = 2; female *i4F*,* n* = 3; female *i4F;p21*‐null, *n* = 4. b. Compilation of dysplasia induction by OSKM in the pancreas of the indicated mice from Figures 1, 2, 3, 4. Compilation of data from Figures 1, 2, 3, 4. *N* values are: *i4F*,* n* = 28; *i4F*;* Il6*‐mutant, *n* = 7; *i4F*;*Ink4a/Arf*‐null, *n* = 9; *i4F;Ink4a*‐null, *n* = 9; *i4F; Arf*‐null, *n* = 16; *i4F;p53*‐null, *n* = 10; *i4F;p53*‐null;*Ink4a/Arf*‐null, *n* = 3; female *i4F*,* n* = 9; female *i4F;p21*‐null, *n* = 10. c. Compilation of reprogramming induction (NANOG+ cells) by OSKM in the pancreas of the indicated mice from Figures 1, 2, 3, 4. Compilation of data from Figures 1, 2, 3, 4. *N* values are: *i4F*,* n* = 23; *i4F*;* Il6*‐mutant, *n* = 4; *i4F*;*Ink4a/Arf*‐null, *n* = 9; *i4F;Ink4a*‐null, *n* = 9; *i4F;Arf*‐null, *n* = 15; *i4F;p53*‐null, *n* = 5; *i4F;p53*‐null;*Ink4a/Arf*‐null, *n* = 2; female *i4F*,* n* = 9; female *i4F;p21*‐null, *n* = 10. d. Correlation between the ‰ NANOG+ cells (assessed by IHC) and the mRNA levels of *Il6* (relative to WT mice and assessed by qRT–PCR). Compilation of all the paired values obtained in this study (*n* = 78). Statistical significance was evaluated by two‐tailed Student's *t* test (*p *<* *.0001) and Pearson correlation (*r *=* *.60). In a–c, all values are expressed as average ± *SEM* Statistical significance was assessed using the unpaired two‐tailed Student's *t* test with Welch's correction: *p *<* *.05, *; *p *<* *.01, **; *p *<* *.001, ***; *p *<* *.0001, ****

The activation of OSKM in vivo triggers not only the reprogramming of a small population of cells, but also induces damage in many other cells (Chiche et al., 2017; Mosteiro et al., 2016). We have previously identified a positive causal connection between damaged cells and reprogramming in tissues upon in vivo OSKM activation (Mosteiro et al., 2016). In this report, we have further explored the in vivo interplay between senescence and reprogramming. To summarize our findings and to facilitate comparison between the different genotypes, we have compiled the key data from Figures 1, 2, 3, 4 into unified panels (Figure 6a–c). For this, we have pooled the groups of control *i4F* male mice in Figures 1a,d, and 4a, thereby increasing the statistical potency of the comparisons.

Importantly, we have confirmed genetically the key role of IL6 in the cross talk between OSKM‐induced senescence and OSKM‐induced reprogramming. We have observed that tissues mutant for IL6 present a reduction in senescence (Figure 6a), which is in agreement with the known role of IL6 in reinforcing senescence in a paracrine and autocrine manner (Kuilman et al., 2008). Consequently, the SASP is also reduced, and this is accompanied by reduced pancreatic dysplasia (Figure 6b) and reprogramming (Figure 6c). In line with this, we have also observed that young female mice present lower levels of OSKM‐induced senescence, pancreatic dysplasia, and reprogramming compared to male mice (Figure 6a–c). This gender difference can be explained by the known negative regulation of NFκB activity and IL6 production by estrogens (Kalaitzidis & Gilmore, 2017; Naugler et al., 2007). We have previously reported that aged mice are more prone to undergo reprogramming than young ones (Mosteiro et al., 2016) and we proposed that this may reflect the higher levels of systemic inflammation and IL6 characteristic of aged mice (Cevenini et al., 2013; Maggio et al., 2006). Estrogen levels are reduced during mouse female aging (Nelson et al., 1981), and indeed, we have also observed that reprogramming was similarly high in aged female and male mice.

To further reinforce the relationship between IL6 and reprogramming, we have represented in the same graph all the paired values of *Il6* mRNA and NANOG+ cells that we have accumulated in this study (*n* = 78 mice with paired data). Importantly, despite the variability of the data, a modest but significant positive association can be detected between the amount of NANOG+ cells and the expression of *Il6* in the pancreas (Figure 6d). IL6 has been implicated in several models of cell dedifferentiation and tissue regeneration (see, e.g., Dubois‐Pot‐Schneider et al., 2014; Gallucci et al., 2000; Lin, Kondo, Ishida, Takayasu & Mukaida, 2003; Schmidt‐Arras & Rose‐John, 2016). Our observations on the critical role of IL6 in the cross talk between damage and reprogramming suggest that the reprogrammable mouse model recapitulates aspects of tissue repair upon injury.

The secretion of IL6 by damaged cells requires a functional *Ink4a/Arf* locus (Mosteiro et al., 2016). *Ink4a/Arf* encodes two different tumor suppressors, p16^Ink4a^ and p19^Arf^, which have separate promoters and reading frames, generating two nonhomologous proteins (Gil & Peters, 2006). p16^Ink4a^ inhibits CDK4, 6/cycD kinases, maintaining the retinoblastoma family proteins in their nonproliferative unphosphorylated form. On the other hand, p19^Arf^ induces cell cycle arrest through MDM2‐mediated stabilization of p53. Here, we show that *Ink4a*‐null mice fail to activate an efficient senescent response upon induction of OSKM (Figure 6a), which results in lower levels of IL6, reduced pancreatic dysplasia (Figure 6b) and lower levels of reprogramming (Figure 6c). In contrast, deletion of *Arf* does not affect neither OSKM‐induced senescence nor in vivo reprogramming (Figure 6a–c). Therefore, *Ink4a*, but not *Arf*, appears to be critical for the interplay between senescence and reprogramming.

We have also demonstrated that OSKM‐induced senescence does not require p53 (Mosteiro et al., 2016). Indeed, tissues lacking *p53* present a stronger damage response upon OSKM activation, leading to increased accumulation of senescent cells and increased reprogramming efficiency (Figure 6a–c). We wondered if *Ink4a* was also critical for senescence and reprogramming in the absence of p53. Triply deficient *p53*/*Ink4a/Arf* mice presented levels of senescence clearly lower than *p53*‐null mice, but higher than in *Ink4a*‐deficient mice (Figure 6a). Interestingly, however, *p53*/*Ink4a/Arf* triply deficient tissues behaved equal to *p53*‐null tissues regarding their high levels of IL6 and reprogramming (Figure 6b,c). Therefore, the absence of *p53* has a dominant effect that renders IL6 production independent of *Ink4a*. In fact, it is known that p53 is a negative regulator of the SASP and its deficiency results in a massive upregulation of the SASP (Coppé et al., 2008).

To shed some light on other known regulators of senescence and reprogramming, we investigated the role of p21. Mice deficient for *p21* present higher levels of OSKM‐induced senescence, SASP production, dysplasia, and reprogramming compared to control *i4F* mice (Figure 6a–c), indicating that it is a barrier for both OSKM‐induced senescence and reprogramming. Of interest, p21 protein levels are notably increased upon OSKM activation, both in wild‐type as well as in *p53*‐null tissues (Mosteiro et al., 2016), and our current data indicate that this is not due to an upregulation of *p21* mRNA levels (Figures 1c,f, 4c and S1). These observations suggest that, upon in vivo OSKM activation, the levels of p21 protein are elevated through post‐transcriptional mechanisms that are known to play a role in p21 regulation (Scoumanne et al., 2011; Warfel & El‐Deiry, 2013; Zhang & Chen, 2008). In this context, it is worth mentioning that the upregulation of p21 during developmentally programmed senescence is also independent of p53 (Muñoz‐Espín et al., 2013; Storer et al., 2013).

All together, we have genetically dissected some of the key players in the cross talk between damage and reprogramming in vivo. These findings may have important implications for the understanding of tissue responses to damage.

## 4 EXPERIMENTAL PROCEDURES

### 4.1 Reprogrammable mice

To generate reprogrammable mice combined with null alleles for each the different tumor suppressor genes and IL6, we used the reprogrammable mouse line known as *i4F‐*B, which carries a ubiquitous doxycycline‐inducible *OSKM* transgene, abbreviated as *i4F*, and inserted into the *Pparg* gene (Abad et al., 2013). Reprogrammable *i4F*‐B mice were crossed with mutant mice for *Ink4a/Arf*,* Ink4a* (Krimpenfort et al., 2001), *Arf* (Kamijo et al., 1997), *p53* (Jacks et al., 1994), *p21* (Brugarolas et al., 1995), or *Il6* (Kopf et al., 1994). Most of these strains were in a pure C57BL/6J.Ola.Hsd genetic background and were compared to *i4F* mice of the same genetic background. The exception was the *i4F;Ink4a*‐null and i4F;*Il6*‐mutant mice that were in a mixed genetic background enriched for C57BL/6J.Ola.Hsd and were compared to *i4F* mice derived from the same parental mice and therefore with similar mixed genetic background.

### 4.2 Animal procedures

Animal experimentation at the CNIO, Madrid, was performed according to protocols approved by the CNIO‐ISCIII Ethics Committee for Research and Animal Welfare (CEIyBA). In general, male mice of 10–13 weeks of age were treated with 0.2 mg/ml doxycycline in the drinking water (supplemented with 7.5% sucrose) for 7 days, except for the *i4F;Ink4a*‐null mice and the corresponding controls that were treated for 10 days. In the case of *i4F;p21*‐null mice and the corresponding *i4F* controls, we used female mice of 10–13 weeks of age. When indicated (Figure 5a), we used old mice of ≥54 weeks of age. Treatment with rIL6 (Abyntek Biopharma, S.L, #AI081) was concomitant to doxycycline treatment and was performed by intraperitoneal injection with 5 μg of rIL6 three times per week during 1 week.

### 4.3 Analysis of mRNA levels

Total RNA was isolated by acid guanidinium thiocyanate–phenol–chloroform extraction from frozen pancreas tissue. Up to 5 µg of total RNA was reverse transcribed into cDNA using iScript™ Advanced cDNA Synthesis Kit for RT–qPCR (Bio‐Rad #172‐5038). Quantitative real‐time PCR was performed using GoTaq^®^ qPCR Master Mix (Promega #A6002) in an ABI PRISM 7700 thermocycler (Applied Biosystem). For input normalization, we used the housekeeping gene *Actin*.

**Table:** 

	Forward primer	Reverse primer
*Actin*	5′‐GGCACCACACCTTCTACAATG‐3′	5′‐GTGGTGGTGAAGCTGTAGCC‐3′
*Arf*	5′‐GCCGCACCGGAGGCACCACACCATCCT‐3′	5′‐TTGAGCAGAAGAGCTGCTACGT‐3′
*Il1a*	5′‐AAGTCTCCAGGGCAGAGAGG‐3′	5′‐CTGATTCAGAGAGAGATGGTCAA‐3′
*Il1b*	5′‐AAAAGCCTCGTGCTGTCG‐3′	5′‐AGGCCACAGGTATTTTGTCG‐3′
*Il1rn*	5′‐TTGTGCCAAGTCTGGAGATG‐3′	5′‐GTTGTGCAGAGGAACCATCC‐3′
*Il6*	5′‐GTTCTCTGGGAAATCGTGGA‐3′	5′‐GGTACTCCAGAAGACCAGAGGA‐3′
*Ink4a*	5′‐TACCCCGATTCAGGTGAT‐3′	5′‐TTGAGCAGAAGAGCTGCTACGT‐3′
*Mmp3*	5′‐CGGGGAGAAGTCCTGTTTTT‐3′	5′‐GGAAGAGATGGCCAAAATGA‐3′
*Pai1*	5′‐CCAACATCTTGGATGCTGAA‐3′	5′‐GCCAGGGTTGCACTAAACAT‐3′
*P53*	5′‐CATCCTTTAACTCTAAGGCCTCATTC‐3′	5′‐AAGATCCGCGGGCGTAA‐3′
*P21*	5′‐CCTTCTCGTGAGACGCTTAC‐3′	5′‐GTGGGTCTGACTCCAGCCC‐3′
*Tnf*	5′‐GCCTCTTCTCATTCCTGCTT‐3′	5′‐CTCCTCCACTTGGTGGTTTG‐3′

### 4.4 Immunohistochemistry

Tissue samples were fixed in 10% neutral‐buffered formalin (4% formaldehyde in solution), paraffin‐embedded, and cut in 3 µm sections, which were mounted in superfrost^®^plus slides and dried. For different staining methods, slides were deparaffinized in xylene and re‐hydrated through a series of graded ethanol until water. Serial sections were stained with hematoxylin and eosin. For immunohistochemistry, an automated immunostaining platform was used (Ventana discovery XT; Roche). Antigen retrieval was first performed with high pH buffer (CC1m; Roche), endogenous peroxidase was blocked, and slides were then incubated with NANOG antibody (Cell Signalling Technology, 8822). After the primary antibody, slides were incubated with the corresponding secondary antibodies and visualization systems (OmniRabbit, Ventana; Roche) conjugated with horseradish peroxidase (Chromomap, Ventana; Roche). Immunohistochemical reaction was developed using 3,30‐diaminobenzidine tetrahydrochloride (DAB) as a chromogen and nuclei were counterstained with hematoxylin. Finally, the slides were dehydrated, cleared, and mounted with a permanent mounting medium for microscopic evaluation. Whole digital slides were acquired with a slide scanner (Mirax Scan; Zeiss), and images captured with the Pannoramic Viewer Software (3DHISTECH). Image analysis and quantification were performed in a completely automated manner using with the AxioVision software package (Zeiss). For each staining, several slides were quantified per mouse (at least three mice per group).

### 4.5 SAβG staining of histological sections

SA*β*G staining was performed in tissue cryosections preserved in OCT freezing medium using the Senescence *β*‐Galactosidase Staining Kit (Cell Signaling, #9860). Briefly, tissue cryosections of 12 µm were fixed at room temperature 5 min with a solution containing 2% formaldehyde and 0.2% glutaraldehyde in PBS, washed three times with PBS, and incubated 48 hrs at 37°C with the staining solution containing X‐gal in N‐N‐dimethylformamide (pH 6.0). Sections were counterstained with nuclear fast red. Image analysis and quantification were performed in a completely automated manner as indicated above for the immunohistochemistry.

### 4.6 Statistical methods

Mice were allocated to their experimental groups according to their predetermined genotype, and therefore, there was no randomization. Investigators were not blinded to the experimental groups. Quantitative PCR data were obtained from independent biological replicates (*n* values correspond to the biological replicates, that is number of mice; technical replicates of the PCR were not considered in the *n* value). Data in Figures 1, 2, 3, 4, 5 are represented as average ± *SD*; data in Figure 6 are represented as average ± *SEM*. Statistical significance was assessed using Student´s *t* test (two‐tailed, unpaired) with Welch´s correction (Pearson correction in the case of Figure 6d).

## CONFLICT OF INTEREST

Authors declare no conflict of interest.

## AUTHOR CONTRIBUTIONS

L.M. performed most of the experiments and participated in the experimental design, data analysis, figure preparation, discussion, and writing; C.P. contributed to most of the experimental work; A. de M. supervised the histological stainings and quantifications, and performed the histopathological evaluations; M.S. contributed to the experimental design, discussion, and writing.

## References

[acel12711-bib-0001] Abad, M. , Mosteiro, L. , Pantoja, C. , Cañamero, M. , Rayon, T. , Ors, I. , … Serrano, M. (2013). Reprogramming in vivo produces teratomas and iPS cells with totipotency features. Nature, 502, 340–345. 24025773 10.1038/nature12586

[acel12711-bib-0002] Banito, A. , Rashid, S. T. , Acosta, J. C. , Li, S. , Pereira, C. F. , Geti, I. , … Gil, J. (2009). Senescence impairs successful reprogramming to pluripotent stem cells. Genes & Development, 23, 2134–2139. 19696146 10.1101/gad.1811609 PMC2751980

[acel12711-bib-0003] Brady, J. J. , Li, M. , Suthram, S. , Jiang, H. , Wong, W. H. , & Blau, H. M. (2013). Early role for IL‐6 signalling during generation of induced pluripotent stem cells revealed by heterokaryon RNA‐Seq. Nature Cell Biology, 15, 1244–1252. 23995732 10.1038/ncb2835 PMC4100556

[acel12711-bib-0004] Brugarolas, J. , Chandrasekaran, C. , Gordon, J. I. , Beach, D. , Jacks, T. , & Hannon, G. J. (1995). Radiation‐induced cell cycle arrest compromised by p21 deficiency. Nature, 377, 552–557. 7566157 10.1038/377552a0

[acel12711-bib-0005] Cevenini, E. , Monti, D. , & Franceschi, C. (2013). Inflamm‐ageing. Current Opinion in Clinical Nutrition and Metabolic Care, 16, 14–20. 23132168 10.1097/MCO.0b013e32835ada13

[acel12711-bib-0006] Chiche, A. , Le Roux, I. , von Joest, M. , Sakai, H. , Aguín, S. B. , Cazin, C. , … Li, H. (2017). Injury‐induced senescence enables in vivo reprogramming in skeletal muscle. Cell Stem Cell, 20, 407–414. 28017795 10.1016/j.stem.2016.11.020

[acel12711-bib-0007] Chuprin, A. , Gal, H. , Biron‐Shental, T. , Biran, A. , Amiel, A. , Rozenblatt, S. , & Krizhanovsky, V. (2013). Cell fusion induced by ERVWE1 or measles virus causes cellular senescence. Genes & Development, 27, 2356–2366. 24186980 10.1101/gad.227512.113 PMC3828521

[acel12711-bib-0008] Coppé, J.‐P. , Patil, C. K. , Rodier, F. , Sun, Y. , Muñoz, D. P. , Goldstein, J. , … Campisi, J. (2008). Senescence‐associated secretory phenotypes reveal cell‐nonautonomous functions of oncogenic RAS and the p53 tumor suppressor. PLoS Biology, 6, e301. 10.1371/journal.pbio.0060301 PMC2592359 19053174

[acel12711-bib-0009] Davaapil, H. , Brockes, J. P. , & Yun, M. H. (2017). Conserved and novel functions of programmed cellular senescence during vertebrate development. Development, 144, 106–114. 27888193 10.1242/dev.138222 PMC5278627

[acel12711-bib-0010] Demaria, M. , Ohtani, N. , Youssef, S. A. , Rodier, F. , Toussaint, W. , Mitchell, J. R. , … Campisi, J. (2014). An essential role for senescent cells in optimal wound healing through secretion of PDGF‐AA. Developmental Cell, 31, 722–733. 25499914 10.1016/j.devcel.2014.11.012 PMC4349629

[acel12711-bib-0011] Dubois‐Pot‐Schneider, H. , Fekir, K. , Coulouarn, C. , Glaise, D. , Aninat, C. , Jarnouen, K. , … Corlu, A. (2014). Inflammatory cytokines promote the retrodifferentiation of tumor‐derived hepatocyte‐like cells to progenitor cells. Hepatology, 60, 2077–2090. 25098666 10.1002/hep.27353

[acel12711-bib-0012] Freund, A. , Orjalo, A. V. , Desprez, P. Y. , & Campisi, J. (2010). Inflammatory networks during cellular senescence: Causes and consequences. Trends in Molecular Medicine, 16, 238–246. 20444648 10.1016/j.molmed.2010.03.003 PMC2879478

[acel12711-bib-0013] Gallucci, R. M. , Simeonova, P. P. , Matheson, J. M. , Kommineni, C. , Guriel, J. L. , Sugawara, T. , & Luster, M. I. (2000). Impaired cutaneous wound healing in interleukin‐6‐deficient and immunosuppressed mice. FASEB Journal, 14, 2525–2531. 11099471 10.1096/fj.00-0073com

[acel12711-bib-0014] Gil, J. , & Peters, G. (2006). Regulation of the INK4b–ARF–INK4a tumour suppressor locus: All for one or one for all. Nature Reviews Molecular Cell Biology, 7, 667–677. 16921403 10.1038/nrm1987

[acel12711-bib-0015] Hanna, J. , Saha, K. , Pando, B. , van Zon, J. , Lengner, C. J. , Creyghton, M. P. , … Jaenisch, R. (2009). Direct cell reprogramming is a stochastic process amenable to acceleration. Nature, 462, 595–601. 19898493 10.1038/nature08592 PMC2789972

[acel12711-bib-0016] Jacks, T. , Remington, L. , Williams, B. O. , Schmitt, E. M. , Halachmi, S. , Bronson, R. T. , & Weinberg, R. A. (1994). Tumor spectrum analysis in p53‐mutant mice. Current Biology, 4, 1–7. 7922305 10.1016/s0960-9822(00)00002-6

[acel12711-bib-0017] Kalaitzidis, D. , & Gilmore, T. D. (2017). Transcription factor cross‐talk: The estrogen receptor and NF‐κB. Trends in Endocrinology and Metabolism, 16, 46–52. 10.1016/j.tem.2005.01.004 15734144

[acel12711-bib-0018] Kamijo, T. , Zindy, F. , Roussel, M. F. , Quelle, D. E. , Downing, J. R. , Ashmun, R. A. , … Sherr, C. J. (1997). Tumor Suppression at the Mouse INK4a Locus Mediated by the Alternative Reading Frame Product p19 ARF. Cell, 91, 649–659. 9393858 10.1016/s0092-8674(00)80452-3

[acel12711-bib-0019] Kawamura, T. , Suzuki, J. , Wang, Y. V. , Menendez, S. , Morera, L. B. , Raya, A. , … Izpisúa Belmonte, J. C. (2009). Linking the p53 tumour suppressor pathway to somatic cell reprogramming. Nature, 460, 1140–1144. 19668186 10.1038/nature08311 PMC2735889

[acel12711-bib-0020] Kopf, M. , Baumann, H. , Freer, G. , Freudenberg, M. , Lamers, M. , Kishimoto, T. , … Köhler, G. (1994). Impaired immune and acute‐phase responses in interleukin‐6‐deficient mice. Nature, 368, 339–342. 8127368 10.1038/368339a0

[acel12711-bib-0021] Krimpenfort, P. , Quon, K. C. , Mooi, W. J. , Loonstra, A. , & Berns, A. (2001). Loss of p16Ink4a confers susceptibility to metastatic melanoma in mice. Nature, 413, 83–86. 11544530 10.1038/35092584

[acel12711-bib-0022] Krizhanovsky, V. , Yon, M. , Dickins, R. A. , Hearn, S. , Simon, J. , Miething, C. , … Lowe, S. W. (2008). Senescence of activated stellate cells limits liver fibrosis. Cell, 134, 657–667. 18724938 10.1016/j.cell.2008.06.049 PMC3073300

[acel12711-bib-0023] Kuilman, T. , Michaloglou, C. , Vredeveld, L. C. W. , Douma, S. , van Doorn, R. , Desmet, C. J. , … Peeper, D. S. (2008). Oncogene‐induced senescence relayed by an interleukin‐dependent inflammatory network. Cell, 133, 1019–1031. 18555778 10.1016/j.cell.2008.03.039

[acel12711-bib-0024] Li, H. , Collado, M. , Villasante, A. , Strati, K. , Ortega, S. , Cañamero, M. , … Serrano, M. (2009). The Ink4/Arf locus is a barrier for iPS cell reprogramming. Nature, 460, 1136–1139. 19668188 10.1038/nature08290 PMC3578184

[acel12711-bib-0025] Lin, Z.‐Q. , Kondo, T. , Ishida, Y. , Takayasu, T. , & Mukaida, N. (2003). Essential involvement of IL‐6 in the skin wound‐healing process as evidenced by delayed wound healing in IL‐6‐deficient mice. Journal of Leukocyte Biology, 73, 713–721. 12773503 10.1189/jlb.0802397

[acel12711-bib-0026] Maggio, M. , Guralnik, J. M. , Longo, D. L. , & Ferrucci, L. (2006). Interleukin‐6 in aging and chronic disease: A magnificent pathway. Journals of Gerontology. Series A, Biological Sciences and Medical Sciences, 61, 575–584. 10.1093/gerona/61.6.575 PMC2645627 16799139

[acel12711-bib-0027] Marion, R. M. , Strati, K. , Li, H. , Murga, M. , Blanco, R. , Ortega, S. , … Blasco, M. A. (2009). A p53‐mediated DNA damage response limits reprogramming to ensure iPS cell genomic integrity. Nature, 460, 1149–1153. 19668189 10.1038/nature08287 PMC3624089

[acel12711-bib-0028] Mosteiro, L. , Pantoja, C. , Alcazar, N. , Marión, R. M. , Chondronasiou, D. , Rovira, M. , … Serrano, M. (2016). Tissue damage and senescence provide critical signals for cellular reprogramming in vivo. Science, 354, aaf4445. 27884981 10.1126/science.aaf4445

[acel12711-bib-0029] Muñoz‐Espín, D. , Cañamero, M. , Maraver, A. , Gómez‐López, G. , Contreras, J. , Murillo‐Cuesta, S. , … Serrano, M. (2013). Programmed cell senescence during mammalian embryonic development. Cell, 155, 1104–1118. 24238962 10.1016/j.cell.2013.10.019

[acel12711-bib-0030] Muñoz‐Espín, D. , & Serrano, M. (2014). Cellular senescence: From physiology to pathology. Nature Reviews Molecular Cell Biology, 15, 482–496. 24954210 10.1038/nrm3823

[acel12711-bib-0031] Naugler, W. E. , Sakurai, T. , Kim, S. , Maeda, S. , Kim, K. , Elsharkawy, A. M. , & Karin, M. (2007). Gender disparity in liver cancer due to sex differences in MyD88‐dependent IL‐6 production. Science, 317, 121–124. 17615358 10.1126/science.1140485

[acel12711-bib-0500] Nelson, J. F. , Felicio, L. S. , Osterburg, H. H. , & Finch, C. E. (1981). Altered profiles of estradiol and progesterone associated with prolonged estrous cycles and persistent vaginal cornification in aging C57BL/6J mice. Biol Reprod. 24(4), 784–794. 7195743 10.1095/biolreprod24.4.784

[acel12711-bib-0032] Ohnishi, K. , Semi, K. , Yamamoto, T. , Shimizu, M. , Tanaka, A. , Mitsunaga, K. , … Yamada, Y. (2014). Premature termination of reprogramming in vivo leads to cancer development through altered epigenetic regulation. Cell, 156, 663–677. 24529372 10.1016/j.cell.2014.01.005

[acel12711-bib-0033] Ritschka, B. , Storer, M. , Mas, A. , Heinzmann, F. , Ortells, M. C. , Morton, J. P. , … Keyes, W. M. (2017). The senescence‐associated secretory phenotype induces cellular plasticity and tissue regeneration. Genes & Development, 31, 172–183. 28143833 10.1101/gad.290635.116 PMC5322731

[acel12711-bib-0034] Schmidt‐Arras, D. , & Rose‐John, S. (2016). IL‐6 pathway in the liver: From physiopathology to therapy. Journal of Hepatology, 64, 1403–1415. 26867490 10.1016/j.jhep.2016.02.004

[acel12711-bib-0035] Scoumanne, A. , Cho, S. J. , Zhang, J. , & Chen, X. (2011). The cyclin‐dependent kinase inhibitor p21 is regulated by RNA‐binding protein PCBP4 via mRNA stability. Nucleic Acids Research, 39, 213–224. 20817677 10.1093/nar/gkq778 PMC3017617

[acel12711-bib-0036] Sharpless, N. E. , & Sherr, C. J. (2015). Forging a signature of in vivo senescence. Nature Reviews Cancer, 15, 397–408. 26105537 10.1038/nrc3960

[acel12711-bib-0037] Storer, M. , Mas, A. , Robert‐Moreno, A. , Pecoraro, M. , Ortells, M. C. , Di Giacomo, V. , … Keyes, W. M. (2013). Senescence is a developmental mechanism that contributes to embryonic growth and patterning. Cell, 155, 1119–1130. 24238961 10.1016/j.cell.2013.10.041

[acel12711-bib-0038] Takahashi, K. , & Yamanaka, S. (2006). Induction of pluripotent stem cells from mouse embryonic and adult fibroblast cultures by defined factors. Cell, 126, 663–676. 16904174 10.1016/j.cell.2006.07.024

[acel12711-bib-0039] Utikal, J. , Polo, J. M. , Stadtfeld, M. , Maherali, N. , Kulalert, W. , Walsh, R. M. , … Hochedlinger, K. (2009). Immortalization eliminates a roadblock during cellular reprogramming into iPS cells. Nature, 460, 1145–1148. 19668190 10.1038/nature08285 PMC3987892

[acel12711-bib-0040] Ventura, A. , Kirsch, D. G. , McLaughlin, M. E. , Tuveson, D. A. , Grimm, J. , Lintault, L. , … Jacks, T. (2007). Restoration of p53 function leads to tumour regression in vivo. Nature, 445, 661–665. 17251932 10.1038/nature05541

[acel12711-bib-0041] Warfel, N. , & El‐Deiry, W. (2013). p21WAF1 and tumourigenesis: 20 years after. Current Opinion in Oncology, 25, 52–58. 23159848 10.1097/CCO.0b013e32835b639e

[acel12711-bib-0042] Xue, W. , Zender, L. , Miething, C. , Dickins, R. A. , Hernando, E. , Krizhanovsky, V. , … Lowe, S. W. (2007). Senescence and tumour clearance is triggered by p53 restoration in murine liver carcinomas. Nature, 445, 656–660. 17251933 10.1038/nature05529 PMC4601097

[acel12711-bib-0043] Zhang, J. , & Chen, X. (2008). Posttranscriptional Regulation of p53 and Its Targets by RNA‐Binding Proteins. Current Molecular Medicine, 8, 845–849. 19075680 10.2174/156652408786733748 PMC2646002

[acel12711-bib-0044] Zhao, Y. , Yin, X. , Qin, H. , Zhu, F. , Liu, H. , Yang, W. , … Deng, H. (2008). Two supporting factors greatly improve the efficiency of human iPSC generation. Cell Stem Cell, 3, 475–479. 18983962 10.1016/j.stem.2008.10.002

