# Supplementary material for: Senescence promotes in vivo reprogramming through p16INK 4a and IL‐6
Source: Aging Cell. 2017 Dec 27;17(2):e12711. doi: 10.1111/acel.12711 (PMC5847859; doi:10.1111/acel.12711)
Supplement: Supplementary file 1 [file ACEL-17-e12711-s001.pdf]

SUPPLEMENTARY FIGURES

Senescence promotes *in vivo* reprogramming through p16^{INK4a} and IL-6

Lluc Mosteiro[1], Cristina Pantoja[1,2], Alba de Martino[1] and Manuel Serrano[1,2,3,*]

Index:

Supplementary Figure S1

Supplementary Figure S2

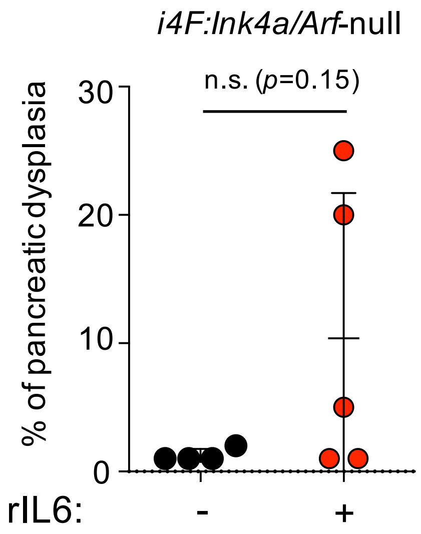

Supplementary Figure S1. Treatment with rIL6 increases *in vivo reprogramming* in *i4F;Ink4a/Arf*-null pancreas

Percentage of dysplasia in the pancreas of *i4F;Ink4a/Arf-null* male mice of 8-10 weeks of age, non-treated (-) and treated (+) with rIL6. Mice were treated with 0.2 mg/ml doxycycline for 7 days and analyzed at the end of the treatment. Treatment with recombinant IL6 (Abyntek Biopharma, S.L, #AI081) was concomitant to doxycycline treatment and was performed by intraperitoneal injection with 5 µg of rIL6 three times during a period of 7 days. Values correspond to % of pancreatic area affected (evaluated blindly), the average ± s.d. are also indicated. Statistical significance was assessed using the unpaired two-tailed Student's t-test with Welch's correction (n.s.: non-significant).

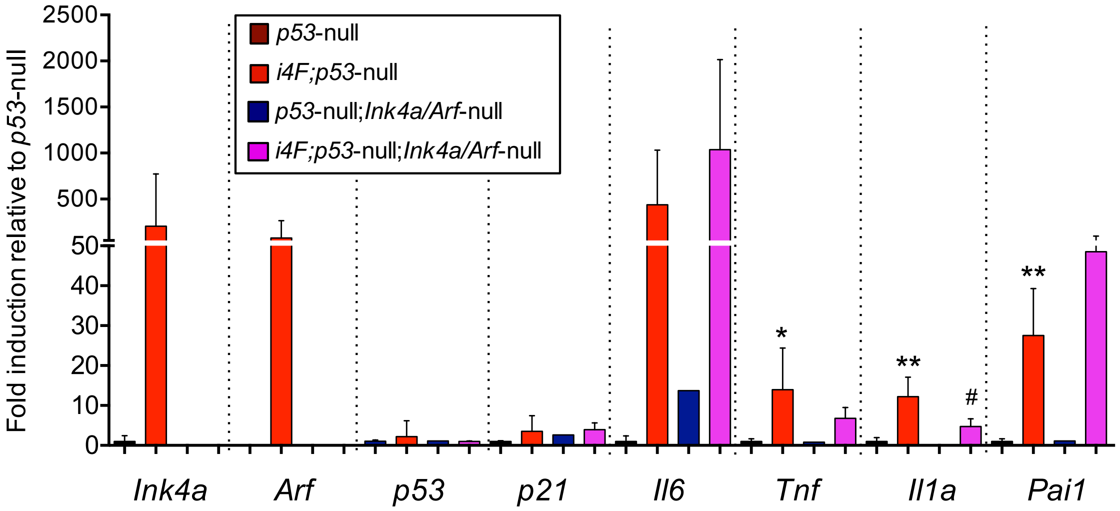

Supplementary Figure S2. Expression levels of senescence genes and SASP cytokines in *i4F;p53*-null;*Ink4a/Arf*-null pancreas

Levels of mRNA of the indicated genes in the pancreas of *p53*-null mice (n=5); *p53*-null;*Ink4a/Arf*-null mice (n=1); *i4F;p53*-null mice (n=6) and *i4F;p53*-null;*Ink4a/Arf*-null mice (n=2). All the mice tested are males of 10-14 weeks of age. Statistical significance compared to *p53*-null controls was assessed using the unpaired two-tailed Student's t-test with Welch's correction: $p<0.05$, *; $p<0.01$, **. Comparisons between *i4F;p53*-null and *i4F; p53*-null;*Ink4a/Arf*-null are indicated in the same manner but using the symbol "#".
